# Image quality and absorbed dose comparison of single‐ and dual‐source cone‐beam computed tomography

**DOI:** 10.1002/acm2.12328

**Published:** 2018-04-17

**Authors:** Hideharu Miura, Shuichi Ozawa, Toshiya Okazue, Atsushi Kawakubo, Kiyoshi Yamada, Yasushi Nagata

**Affiliations:** ^1^ Hiroshima High‐Precision Radiotherapy Cancer Center Hiroshima Japan; ^2^ Department of Radiation Oncology Institute of Biomedical & Health Science Hiroshima University Hiroshima Japan

**Keywords:** absorbed dose, dual‐source cone‐beam computed tomography, image quality, image‐guided radiation therapy

## Abstract

**Purpose:**

Dual‐source cone‐beam computed tomography (DCBCT) is currently available in the Vero4DRT image‐guided radiotherapy system. We evaluated the image quality and absorbed dose for DCBCT and compared the values with those for single‐source CBCT (SCBCT).

**Methods:**

Image uniformity, Hounsfield unit (HU) linearity, image contrast, and spatial resolution were evaluated using a Catphan phantom. The rotation angle for acquiring SCBCT and DCBCT images is 215° and 115°, respectively. The image uniformity was calculated using measurements obtained at the center and four peripheral positions. The HUs of seven materials inserted into the phantom were measured to evaluate HU linearity and image contrast. The Catphan phantom was scanned with a conventional CT scanner to measure the reference HU for each material. The spatial resolution was calculated using high‐resolution pattern modules. Image quality was analyzed using ImageJ software ver. 1.49. The absorbed dose was measured using a 0.6‐cm^3^ ionization chamber with a 16‐cm‐diameter cylindrical phantom, at the center and four peripheral positions of the phantom, and calculated using weighted cone‐beam CT dose index (CBCTDI_w_).

**Results:**

Compared with that of SCBCT, the image uniformity of DCBCT was slightly reduced. A strong linear correlation existed between the measured HU for DCBCT and the reference HU, although the linear regression slope was different from that of the reference HU. DCBCT had poorer image contrast than did SCBCT, particularly with a high‐contrast material. There was no significant difference between the spatial resolutions of SCBCT and DCBCT. The absorbed dose for DCBCT was higher than that for SCBCT, because in DCBCT, the two x‐ray projections overlap between 45° and 70°.

**Conclusions:**

We found that the image quality was poorer and the absorbed dose was higher for DCBCT than for SCBCT in the Vero4DRT.

## 1 INTRODUCTION

Cone‐beam computed tomography (CBCT) imaging for image‐guided radiotherapy (IGRT) uses soft tissue and volumetric anatomic imaging information for higher geometric accuracy of radiotherapy delivery.^1^ Stereotactic body radiotherapy (SBRT), intensity‐modulated radiotherapy (IMRT), and volumetric‐modulated arc radiotherapy (VMAT) techniques require precisely delivered doses to the planning target volume (PTV). CBCT with a kilovolt (kV) source and a flat‐panel detector (FPD) mounted onto the gantry of a linear accelerator is the common configuration used for IGRT. The image quality of CBCT is crucial for accurate localization in the patient.^2^ Several studies on image quality and absorbed dose of commercially available CBCT have been reported.^3^, ^4^, ^5^, ^6^, ^7^, ^8^ Most of the investigations were on single‐source CBCT (SCBCT) imaging.

Vero4DRT (Mitsubishi Heavy Industries, Ltd., Hiroshima, Japan, and Brainlab, Munich, Germany) is a unique image‐guided radiotherapy system comprising two imaging units aligned at ±45° relative to a megavoltage (MV) beam axis. Each imaging unit consists of a kV X ray tube and a FPD. Miura et al.^6^ reported the image quality assurance (QA) for the Vero4DRT system with SCBCT. In SCBCT, it takes approximately 30 s to acquire the projection data using a 215° rotation because the rotation speed is limited to 7°/s. The Vero4DRT is now available with dual‐source CBCT (DCBCT), in which it takes approximately 15 s to acquire the projection data using a 115° rotation, making it very useful for reducing the treatment time. In addition, DCBCT might reduce motion artifacts. DCBCT also plays a large role in some 4D‐CBCT techniques, which may benefit from a dual‐source technique.^9^

Two important issues need to be addressed when using DCBCT. First, how does the image quality of DCBCT compare with that of SCBCT? Second, does DCBCT increase the patient dose compared with SCBCT? The purpose of CBCT is to provide a volumetric image for patient positioning for radiotherapy; thus, it is important to study the dose–image quality tradeoffs. However, no information is available on the imaging performance of a commercial DCBCT.

In this study, we evaluated the performance of a DCBCT, by comparing it with a SCBCT. Both were used in a Vero4DRT. The American Association of Physicists in Medicine (AAPM) Task Group (TG) 142 recommends several QA values for imaging by IGRT.^10^ We focused on image uniformity, Hounsfield unit (HU) linearity, image contrast, spatial resolution, and absorbed dose for CBCT.

## 2 MATERIALS AND METHODS

### 2.A Vero4DRT

**Figure 1:**
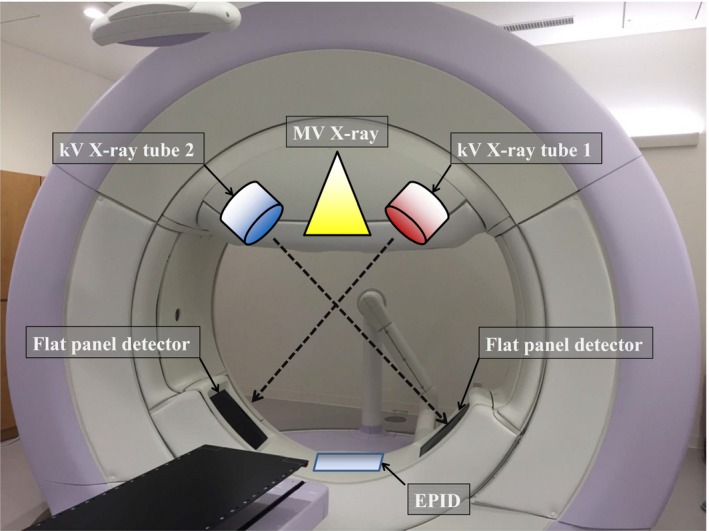
Vero4DRT system with two kV imaging systems, each consisting of a kV x‐ray tube and flat panel detector (FPD) aligned at ±45° relative to the MV x‐ray beam axis. The diameter of the O‐ring structure is about 350 cm.

The characteristics of the Vero4DRT system were published previously^11^ (Fig. 1). The gantry is extremely rigid owing to its O‐ring shape. The Vero4DRT system has two kV x‐ray imaging subsystems attached to the O‐ring and two FPDs at 45° with respect to the MV beam axis. The CBCT images are acquired using kV x‐ray tubes by rotating the gantry.

**Figure 2:**
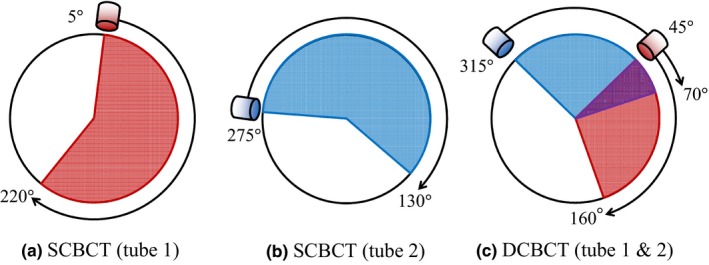
Image acquisition using SCBCT (a) tube 1 rotated 215° between 5° to 220° (red) and (b) tube 2 rotated 215° between 275° to 130° (blue). (c) Image acquisition using DCBCT requires both kV x‐ray tubes be rotated 115°(tube1: 315°–70°, tube2: 45°–160°). The purple area indicates the overlap angle (45°–70°).

To acquire a set of SCBCT images, a kV X‐ray source is rotated 215° clockwise (CW) or counterclockwise (CCW) [Fig. 2(a,b)]. To acquire a set of DCBCT images, both kV x‐ray sources are rotated 115° CW or CCW [Fig. 2(c)]. In our study, SCBCT was performed using only one tube and DCBCT was performed using the two tubes simultaneously. We used only CW rotation because there would be no difference in image quality between CW and CCW rotations.^6^ For image acquisition, we used the following x‐ray parameters: tube voltage = 120 kV, tube current = 200 mA, and pulse width = 10 ms. In the Vero4DRT system, the scan field of view (FOV) is a cylinder 200 mm in diameter and 150 mm long. A 512 × 512‐voxel reconstruction matrix was used to reconstruct the FOV of 200 cm, resulting in a pixel size of 0.39 mm. With the Vero4DRT, the slice thickness can be selected from the range of 0.5–3.0 mm. In this study, we selected a slice thickness of 3.0 mm for image quality evaluation. The vendor performed the gain and offset corrections for the detector.

### 2.B Image quality

The image uniformity, HU linearity, spatial resolution, and image contrast were evaluated using a Catphan 504 CT phantom (The Phantom Laboratory, Greenwich, NY, USA), a well‐established and validated QA tool that incorporates several modules for CT QA.^12^ Several studies on the characteristics of cross‐vendor CBCT have been conducted using the Catphan phantom.^3^, ^4^

We setup the Catphan phantom at the end of the treatment couch. The in‐room laser system used the alignment marks on the surface of the phantom to position it for each imaging device. The position alignment of the phantom was checked using four wire ramps that increased to a 23° angle from the base to the top of the module. The image quality of all the regions of interest (ROI) was statistically analyzed using ImageJ software ver. 1.49 (National Institutes of Health, Bethesda, MD, USA, http://rsb.info.nih.gov/ij). Five CBCT images were acquired and their average and standard deviation were calculated for each evaluation of image quality.

#### 2.B.1 Image uniformity

**Figure 3:**
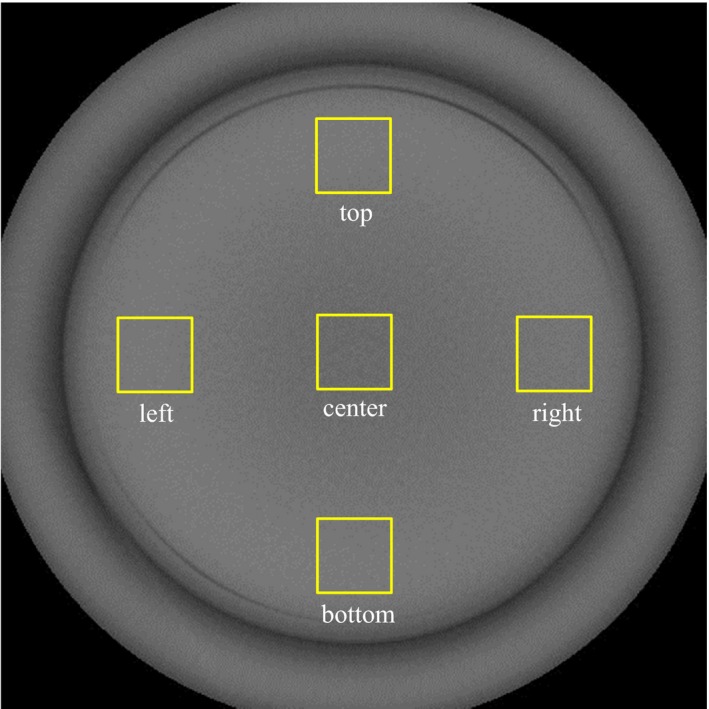
DCBCT image of the uniformity phantom (CTP 486). Five ROIs (center, top, left side, bottom, and right side) on the image were used to evaluate image uniformity.

The uniformity module CTP 486, with a uniform disk, was used to assess image uniformity. Five 3.0‐cm × 3.0‐cm ROIs at the center and the top, bottom, left, and right peripheral positions of the image were assessed (Fig. 3). Image uniformity was calculated using the following equation:

(1) $$\text{Image uniformity}=|{\overset{¯}{\mathrm{CT}}}_{\mathrm{ROI},\mathrm{peripheral}}-{\overset{¯}{\mathrm{CT}}}_{\mathrm{ROI},\mathrm{center}}|$$

where ${\overset{¯}{\mathrm{CT}}}_{\mathrm{ROI},\mathrm{peripheral}}$ and ${\overset{¯}{\mathrm{CT}}}_{\mathrm{ROI},\mathrm{center}}$ are the mean pixel value of the ROIs at the four peripheral positions and the center, respectively.

#### 2.B.2 HU linearity

**Figure 4:**
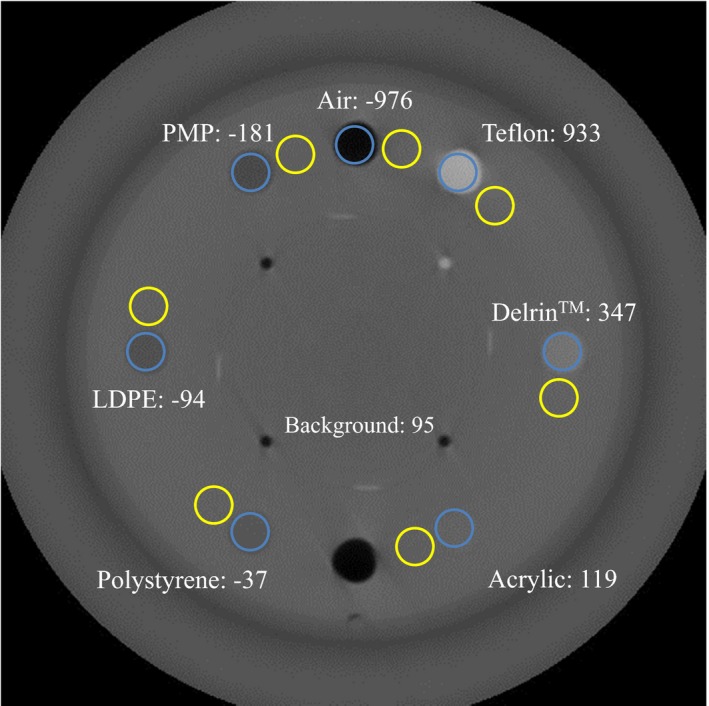
DCBCT image of the HU reproducibility module (CTP 404) with seven inserts of materials with different densities. The blue circles are the ROIs inside the inserts and the yellow circles are the ROIs surrounding the inserts and are used to measure background. Numbers are the HU values from diagnostic CT.

The slice width, sensitometry, and pixel size module CTP 404, consisting of seven materials with different densities [air, PMP (polymethylpentene), LDPE (low‐density polyethylene), polystyrene, acrylic, Delrin^®^ (polyoxymethylene), and Teflon™ (polytetrafluoroethylene)], was used to assess HU linearity (Fig. 4). The mean HU value for each material was measured within a 1.0‐cm‐diameter circle. The Catphan phantom was scanned with an Optima CT580W scanner (GE Healthcare, Chicago, IL, USA) to measure the reference HU of each material. The scanning parameters were as follows: tube voltage = 120 kV, FOV = 200 mm, collimation = 1.25 mm, matrix = 512 pixels × 512 pixels, and slice thickness = 2.5 mm. The exposure was fixed at 200 mAs per frame.

#### 2.B.3 Image contrast

We used the CTP 404 module, described above, to assess the image contrast, which was calculated using the following equation:

(2) $$\text{image contrast}=|{\overset{¯}{P}}_{\mathrm{ROI},\mathrm{insert}}-{\overset{¯}{P}}_{\mathrm{ROI},\mathrm{background}}|$$

where ${\overset{¯}{P}}_{\mathrm{ROI},\mathrm{insert}}$ is the mean pixel value in a circled ROI inside an insert and ROI, background is the mean pixel value of the background of the contrast module, respectively.

#### 2.B.4 Spatial resolution

The high‐resolution module CTP 528 has a high‐resolution pattern of 1 through 21 line pairs per centimeter (Lp/cm). High‐contrast resolution was calculated using the method reported by Droege et al.^13^ In that method, the practical modulation transfer function (pMTF) curve is calculated by measuring the standard deviation of the pixel values in each individual pattern in the cyclic bar pattern image. To assess the spatial resolution quantitatively, 50% and 10% values were calculated from the pMTF curve data.

#### 2.B.5 Absorbed dose

Dose measurements were performed using methodology adapted from that outlined in AAPM Task Group Report No. 111.^14^ The CBCT absorbed dose was measured using a 0.6‐cm^3^ ionization chamber (Radcal, Monrovia, CA, USA) and a poly(methyl methacrylate) (PMMA) cylindrical phantom (Radcal), 16 cm in diameter and 15 cm long with a density of 1.19 g/cm^3^. The midpoint of the cylindrical cavities, located at the center and four peripheral positions in the phantom, corresponded to the in‐room laser system. The ionization chamber was inserted at the center and the four peripheral holes of the cylindrical phantom, with the mechanical isocenter at the center of the phantom. Because CBCT performed with the Vero4DRT involves a partial rotation of the gantry to acquire images, the dose at each of the peripheral measurement points is different. The average absorbed dose was calculated by analogy to the weighted cone‐beam CT dose index (CBCTDI_w_) 14 using the following equation:

(3) $${\text{CBCTDI}}_{w}=\left(1/3\right)D_{\mathrm{center}}+\left(2/3\right)D_{\mathrm{peripheral}}$$

where *D*_center_ is the central axis dose and *D*_peripheral_ is the average peripheral dose of the scanning phantom.^15^ The other holes of the phantom were filled in with PMMA rods to avoid affecting the measurements. The absorbed dose was measured five times for each position of the ionization chamber.

## 3 RESULTS

### 3.A Image uniformity

Table 1 presents the HU values measured at the center and peripheral positions of the phantom. The maximum differences in the HU between the center and the peripheral positions for SCBCT (tube 1/tube 2) and DCBCT were 6.8/10.9 and 31.1 HU, respectively. The image uniformity of DCBCT was poorer than that of SCBCT.

**Table 1:** Performance of SCBCT and DCBCT in image uniformity using a uniformity module

	SCBCT (tube 1)	SCBCT (tube 2)	DCBCT
Center	−53.5 ± 6.3	−43.7 ± 6.0	−174.3 ± 5.0
Top	−61.2 ± 8.1	−59.6 ± 10.1	−143.2 ± 6.8
Left	−60.9 ± 7.4	−54.5 ± 8.2	−144.2 ± 6.5
Bottom	−60.9 ± 7.6	−60.0 ± 8.8	−144.6 ± 6.8
Right	−60.2 ± 9.6	−62.3 ± 9.6	−153.3 ± 5.7
Image uniformity	7.4	15.4	28.0

All values are average values ± standard deviation (SD) and are expressed in HU.

### 3.B HU linearity

**Figure 5:**
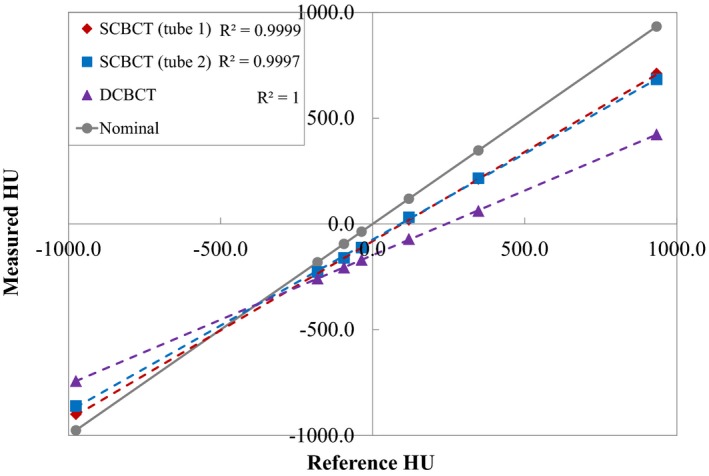
Reference HU values as a function of measured HU values using SCBCT and DCBCT. Dashed lines are linear regression fits of the data and *R* ^2^ is the coefficient of determination. The solid gray line indicates where the reconstructed HU is equal to the reference HU.

Figure 5 shows the plot of the reference HU values of the Catphan phantom inserts as a function of the HU values measured in the ROIs in the SCBCT and DCBCT images. The measured HU values had a linear relationship with the reference HUs, with a coefficient of determination (*R* ^2^) >0.9997. The measured HU values for SCBCT were closer to the reference HU values than those for DCBCT. The largest differences between the reference and the measured HU values occurred for Teflon; the difference in the HU values obtained using SCBCT (tube 1/tube 2) and DCBCT were 221.9/249.6 and 510.4 HU, respectively.

### 3.C Image contrast

**Figure 6:**
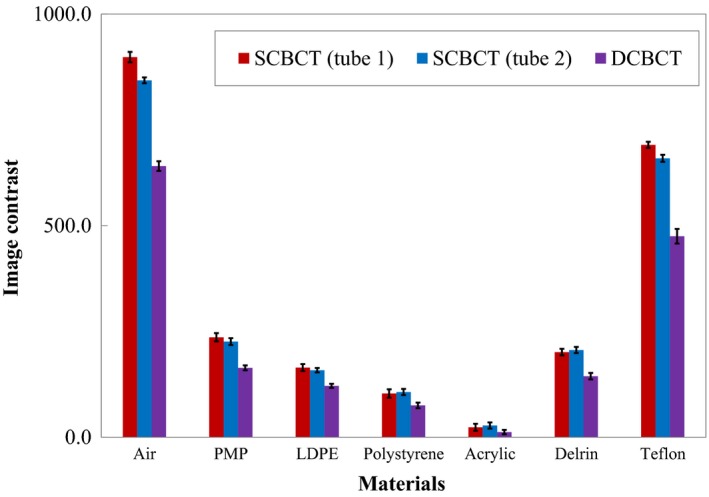
Comparison between image contrasts obtained using SCBCT and DCBCT of the seven insert materials of different densities. DCBCT yielded poorer image contrast than did SCBCT for all materials (low image contrast value indicates poor contrast). Error bars correspond to 1 SD for the mean value of five measurements.

Figure 6 compares the image contrasts obtained using SCBCT and DCBCT of the seven inserted materials of different densities. The image contrast obtained with DCBCT was poorer than that obtained with SCBCT for all materials. In particular, the differences between SCBCT (tube 1/tube 2) and DCBCT image contrast values were largest for the high‐contrast materials air and Teflon. The image contrast values for air obtained using SCBCT (tube 1/tube 2) and DCBCT were 898.2/843.3 and 640.8 HU, respectively, and those for Teflon were 690.8/659.1 and 475.0 HU, respectively. The image contrast values for the low‐contrast material polystyrene obtained using SCBCT (tube 1/tube 2) and DCBCT were 103.3/107.0 and 75.3 HU, respectively.

### 3.D Spatial resolution

Table 2 shows the spatial resolution results for the 50% and 10% MTF obtained with SCBCT (tube 1/tube 2) and DCBCT. There was no significant difference in spatial resolution between SCBCT and DCBCT.

**Table 2:** Performance of SCBCT and DCBCT in MTF using a high‐contrast‐resolution module

	50% MTF (mm^−1^)	10% MTF (mm^−1^)
SCBCT (tube 1)	0.39 ± 0.01	0.77 ± 0.01
SCBCT (tube 2)	0.37 ± 0.02	0.75 ± 0.02
DCBCT	0.37 ± 0.01	0.76 ± 0.01

MTF, modulation transfer function.

All values are average values ± standard deviation (SD).

### 3.E Absorbed dose

Table 3 presents the absorbed dose for SCBCT and DCBCT obtained using a 16‐cm‐diameter cylindrical phantom. The CBCTDI_w_ values of SCBCT (tube 1/tube 2) and DCBCT were 54.3/59.4 and 56.4 mGy, respectively. The CBCTDI_w_ value of DCBCT was 13.5% and 3.8% higher than those of SCBCT (tube 1/tube 2).

**Table 3:** Absorbed doses and CBCTDI_w_ values obtained using 16‐cm‐diameter cylindrical phantom

	SCBCT (tube 1)	SCBCT (tube 2)	DCBCT
Center	47.1 ± 0.2	50.9 ± 0.1	53.3 ± 0.1
Top	50.1 ± 0.1	96.5 ± 0.1	89.3 ± 0.1
Left	25.8 ± 0.1	51.4 ± 0.1	34.2 ± 0.1
Bottom	66.4 ± 0.1	29.6 ± 0.1	42.0 ± 0.1
Right	89.3 ± 0.1	77.0 ± 0.1	98.0 ± 0.1
CBCTDI_w_	54.3 ± 0.1	59.4 ± 0.1	61.7 ± 0.1

All values are expressed in mGy.

All values are average values ± standard deviation (SD).

## 4 DISCUSSION

We compared the image quality and absorbed dose for the first commercial DCBCT, in the Vero4DRT image‐guided radiotherapy system, with those for SCBCT. The CBCT of the Vero4DRT system cannot rotate a full 360°, which leads to lower image uniformity. The image uniformity of DCBCT is worse than that of SCBCT. With DCBCT, the detector may detect the photons scattered by the object, resulting in degradation of the image quality.^16^, ^17^ Engel et al. 18 used a Monte Carlo method to simulate photon scatter in the SCBCT and DCBCT. With SCBCT, the image uniformity obtained with tube 2 was worse than that of tube 1, which implies that the two SCBCT systems have slightly different detector responses. Using the projection data from two SCBCT systems, with their different responses, in one coordinate system led to the degradation of image uniformity in the DCBCT system. Thus, the poor image uniformity of DCBCT is attributed not only to the scattered radiation but also to the difference in detector response. In addition, the heel effect of the x‐ray tube, whereby the intensity of the x‐ray beam emitted by the tube depends on the direction of emission, could also affect image uniformity.

No significant degradation in HU linearity for DCBCT was observed when compared to that for SCBCT. The HU linearity of both CBCTs differed from the reference HU. The Teflon HU value obtained via DCBCT had the largest difference with respect to the reference HU, which is attributed to cross‐scatter.^16^ The HU linearity of the Vero4DRT system differs from that of conventional CT. However, this is not a problem because the Vero4DRT system does not use the concept of adaptive radiotherapy treatment planning with CBCT. CBCT of the Vero 4DRT system does not use a calibration method that matches the gradation degree with known parameters such as bone density and air density. The images acquired using DCBCT have poorer contrast than those acquired using SCBCT, probably because the cross‐scatter generated by DCBCT is higher than that by SCBCT. Even for a low‐contrast material (e.g., acrylic), DCBCT‐acquired images had poorer contrast than SCBCT‐acquired images. Acrylic had a low image contrast because acrylic and the background material (PMMA) were the same. The required contrast resolution depends on the anatomical region. The user should optimize the imaging parameters to improve the image contrast. However, increasing the kV/mA ratio increases the exposure dose. In the Vero4DRT, the projections in DCBCT overlap between 45° and 70° [see Fig. 2(c)]; thus, the absorbed dose is higher than that for SCBCT for the same kV/mA. Although the dose from image guidance is small compared with the uncertain dose delivered in therapy, based on standard radiation safety principles, the imaging dose of DCBCT at a minimum should be identical to that of SCBCT. The PMMA phantom used in our study was 16 cm in diameter and 15 cm long. CT dosimetry systems need radiation absorption and scattering phantoms sufficiently long to accommodate scanning lengths related to cumulative dose equilibrium, as described in AAPM Task Group Report No. 111.^14^ The absorbed dose in a 16‐cm‐diameter PMMA phantom is close to that in a 20‐cm‐diameter cylinder of water.^19^

Several authors proposed techniques to reduce the cross‐scatter and improve the image quality for DCBCT [16‐18, 20]. Giles et al.^17^ proposed that almost all cross‐scatter effects can be removed by interleaved acquisition, which can be achieved at the same angular sampling rate by either doubling the data acquisition rate or halving the rotation speed. In another study, a bowtie filter was used to reduce the scatter‐to‐primary radiation ratio and improve image quality.^20^ Because of the limitation of the hardware used in scatter‐reduction methods, Zhu et al.^16^ proposed reducing the cross‐scatter effects using post‐processing techniques. In the Vero4DRT system, no cross‐scatter correction method, including an effective acquisition technique, bowtie filter, or post‐processing technique, is implemented.

**Figure 7:**
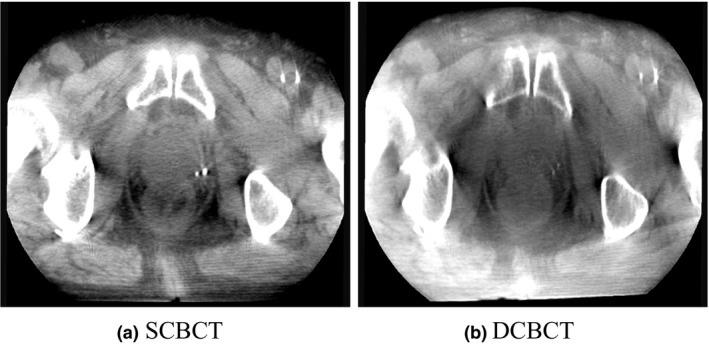
Axial images of the pelvis region obtained using (a) SCBCT and (b) DCBCT.

A CBCT scan can be acquired over a 360° rotation at a maximum angular speed of one rotation per minute. In the Vero4DRT or other on‐board SCBCT systems, acquisition of a CBCT half‐scan using a single kV source takes approximately 30 s, which makes a breath‐hold CT scan difficult to implement. Future studies should investigate the effect of DCBCT on reducing motion artifacts. Data from a phantom may not represent the final say in image quality with respect to clinical patient data because a phantom is relatively uniform, unlike a patient. Figure 7 shows axial images of the pelvic region obtained with SCBCT and DCBCT. A CBCT correction algorithm for clinical use is necessary to improve the DCBCT image quality.^21^ We will further investigate the performance of DCBCT in other imaging regions (e.g., thorax and abdomen).

## 5 CONCLUSIONS

In this study, we evaluated the image quality and absorbed dose for DCBCT by comparing them with the corresponding values for SCBCT, using the Vero4DRT. Compared with SCBCT, DCBCT had slightly reduced image uniformity. A strong linear correlation existed between the mean HU values in the ROIs obtained by DCBCT and the reference HU, although the linear regression slope was different from that of conventional CT. The image contrast with DCBCT, particularly for extreme‐contrast material, was worse than that with SCBCT, even though the absorbed dose was higher.

## CONFLICT OF INTEREST

No conflicts of interest.
